# Supplementary material for: A rapid inducible RNA decay system reveals fast mRNA decay in P-bodies
Source: Nat Commun. 2024 Mar 28;15:2720. doi: 10.1038/s41467-024-46943-z (PMC10979015; doi:10.1038/s41467-024-46943-z)
Supplement: Supplementary file 1 — Supplementary Information [file 41467_2024_46943_MOESM1_ESM.pdf]

## Supplementary Information

# A rapid inducible RNA decay system reveals fast mRNA decay in P-bodies

Lauren A. Blake[1,2], Leslie Watkins[1,2], Yang Liu[1,2,3], Takanari Inoue[2,4], Bin Wu[1,2,5,†]

[1]Department of Biophysics and Biophysical Chemistry, Johns Hopkins University School of Medicine, Baltimore, MD 21205, USA.

[2]The Center for Cell Dynamics, Johns Hopkins University School of Medicine, Baltimore, MD 21205, USA.

[3]Current address: Department of Biochemistry, University of Utah, Salt Lake City, UT 84112, USA.

[4]Department of Cell Biology, Johns Hopkins University School of Medicine, Baltimore, MD 21205, USA.

[5]The Solomon H Snyder Department of Neuroscience, Johns Hopkins University School of Medicine, Baltimore, MD 21205, USA.

† Corresponding Author: bwu20@jhmi.edu

## Contents

**Supplementary Figure 1**

Tethering SMG7C to 24xMBS produced highest knockdown efficiency.

**Supplementary Figure 2**

RIDR is fast, specific, and inducible on endogenous ACTB-MBS gene.

**Supplementary Figure 3**

Induced RNA granules colocalize with P-body marker DDX6 and not stress granule marker G3BP1.

**Supplementary Figure 4**

ACTB-ORF and MBSv6 RNA are recruited to P-bodies.

**Supplementary Figure 5**

SMG7C does not localize to P-bodies in steady state conditions.

**Supplementary Figure 6**

Translation inhibitors have different effects on RNA decay dynamics.

**Supplementary Figure 7**

Fitting to kinetic model with no assumptions shows similar results as Assumption III: $k_L$=0.

**Supplementary Figure 8**

Knockdown of DDX6 and XRN1 RNA is efficient.

**Supplementary Figure 9**

Arsenite stress alone does not result in RNA accumulation in P-bodies.

## Supplementary Figure 1

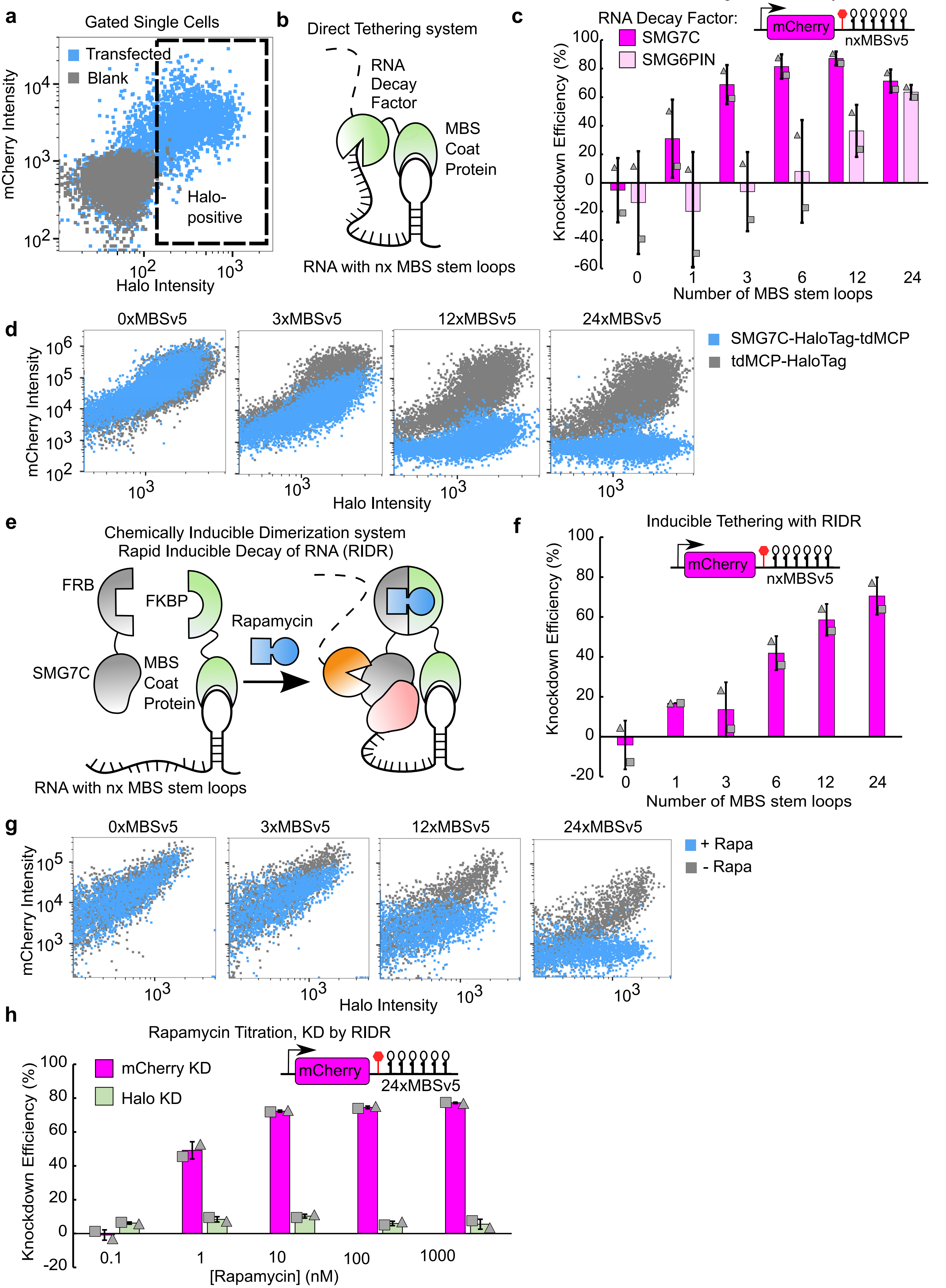

**Supplementary Figure 1:** ***Tethering SMG7C to 24xMBS produced highest knockdown efficiency*** **a)** Gating strategy for all flow cytometry experiments demonstrating how single HEK293T cells were gated. Representative flow data of cells that were co-transfected with mCherry-nxMBS and fusions of HaloTag-tdMCP with an RNA decay factor or CID protein (blue) or nothing (gray). Cells were transfected for 12-16 hours prior to preparation for flow cytometry. HaloTag-positive cells (dashed black box) were selected for the quantification of mCherry knockdown for each condition relative to a negative control. **b)** Schematic of the direct tethering of a general RNA decay factor to an MBS by fusing the RNA decay factor to the tandem MS2 coat protein (tdMCP) **c)** HEK293T cells were transiently transfected with mCherry-nxMBS (where n = 0, 1, 3, 6, 12, 24) and SMG7C-HaloTag-tdMCP or SMG6PIN-HaloTag-tdMCP constructs. HaloTag was labelled with JF503 Halo-ligand. The fluorescence of single cells was measured by flow cytometry. The knockdown efficiency was calculated by measuring the geometric mean of mCherry and normalizing this value to negative control tdMCP-HaloTag without decay factor. **d)** Raw flow cytometry data for mCherry reporters tagged with 0, 3, 12, and 24 MBS Stem loops, comparing SMG7C-HaloTag-tdMCP (blue) vs tdMCP-HaloTag negative control (gray) 40000-60000 HaloTag-positive cells were quantified per condition. **e)** Schematic of the inducible mRNA decay system using FRB/FKBP CID induced by rapamycin ligand. SMG7C-fused FRB is tethered to target RNA with MBS by FKBP-tdMCP to cause its degradation. **f)** Knockdown efficiencies of mCherry-nxMBS were quantified from flow cytometry experiments using the inducible system shown in (c). The knockdown efficiency for each condition was calculated with respect to itself when Rapa was not added. **g)** Raw flow cytometry data for mCherry reporters tagged with 0, 3, 12, and 24 MBS, comparing RIDR with (blue) and without (gray) 100nM Rapa induction. Rapa was added at the same time as transfection. **h)** Titration of Rapa concentration (ranging from 0.1nM to 1000nM) for RIDR experiment . The knockdown efficiency for each condition was calculated with respect to itself when Rapa was not added. 5000-10000 HaloTag-positive cells were quantified per condition. Data are presented as mean values across 2 biological replicates. Error bars represent the standard deviation. Source data are provided as a Source Data file.

# Supplementary Figure 2

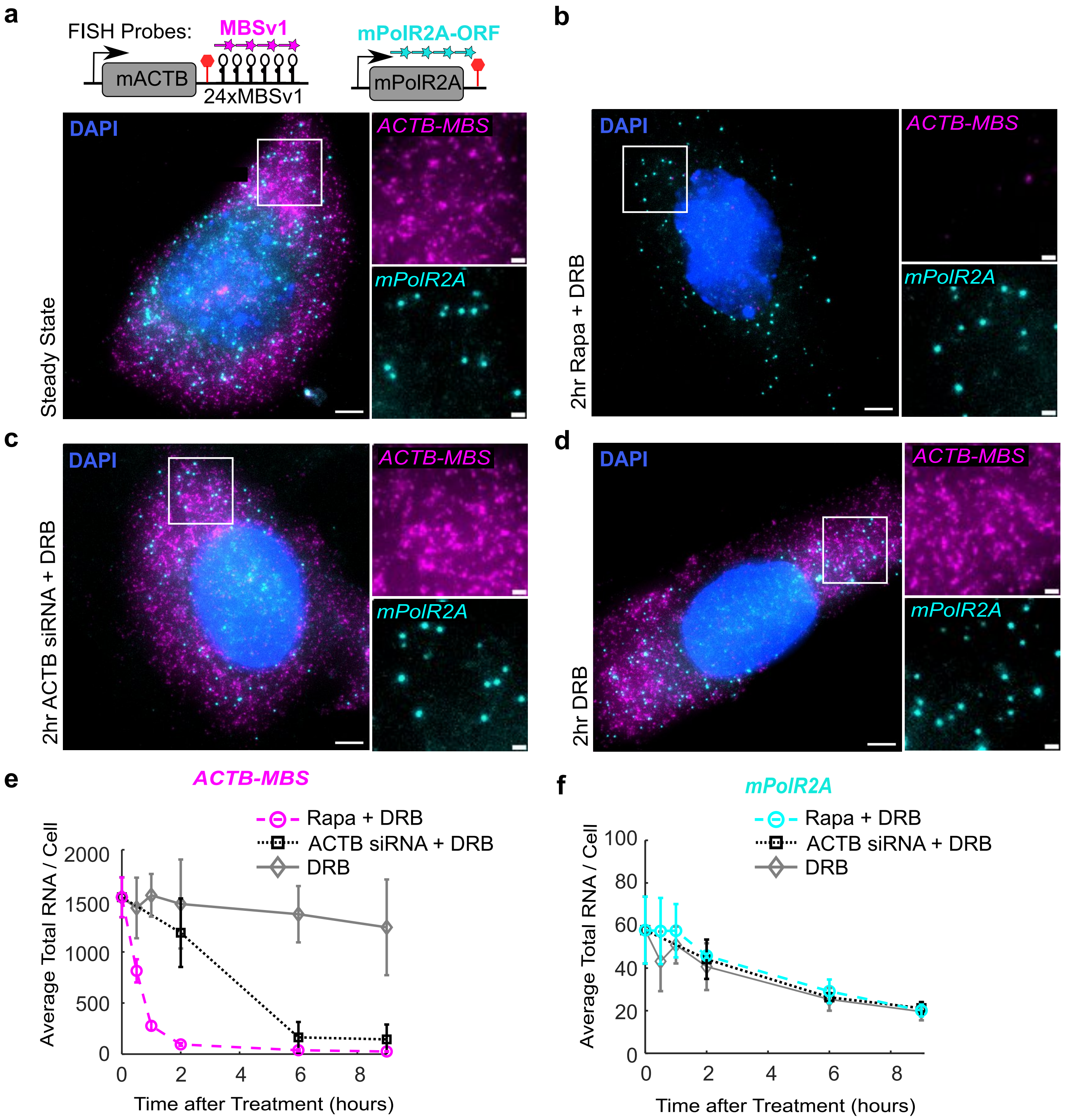

**Supplementary Figure 2: *RIDR is fast, specific, and inducible on endogenous ACTB-MBS gene.*** **a-d)** Representative smFISH images of ACTB-MBS MEF cells stably expressing RIDR in steady state conditions **(a)**, after 2 hours Rapa + DRB treatment **(b)**, after 2 hours ACTB siRNA treatment **(c)**, and after 2 hours of DRB treatment alone **(d)**. The white boxes were enlarged on the right. *ACTB-MBS* FISH: magenta; *mPolR2A* FISH: cyan; DAPI: blue. Scale bars: 5 µm for original and. 1 µm for zoomed images. **e-f)** Quantification of time-resolved two-color smFISH experiment over 9-hours after induction with different treatments. The number of transcripts for *ACTB-MBS* **(e)** and *mPolR2A* **(f)** were counted in the same cells for all time points. Rapa + DRB: circles; ACTB siRNA + DRB: squares; DRB alone: diamonds. Error bars represent standard deviation of the means of 3-4 biological replicates. 125-253 cells were quantified per condition across replicates (the precise number of cells per condition per replicate are given in the source data).Source data are provided as a Source Data file.

## Supplementary Figure 3

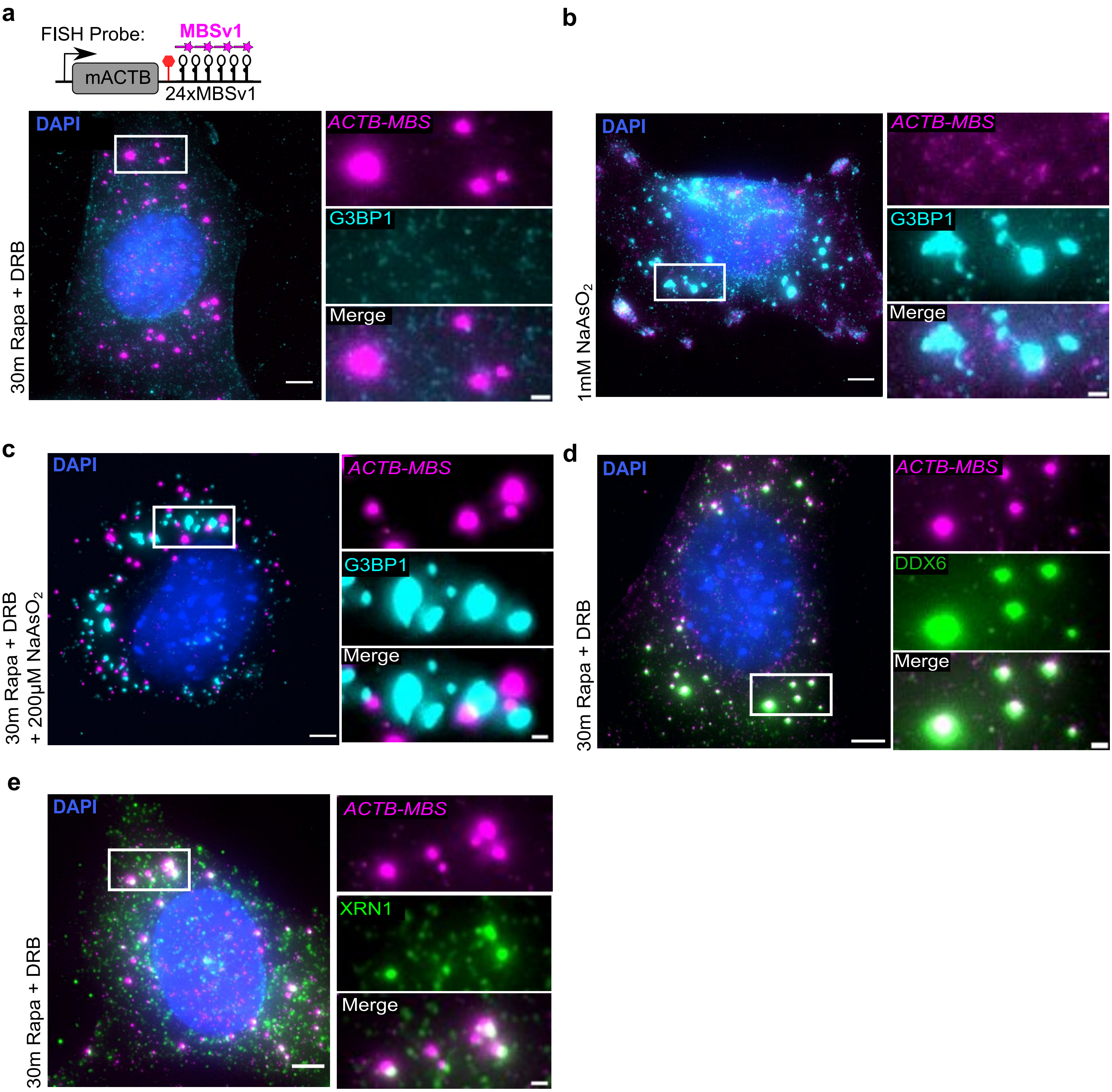

**Supplementary Figure 3:** ***Induced RNA granules colocalize with P-body markers DDX6 and XRN1 but not stress granule marker G3BP1.*** smFISH-IF experiments were conducted on ACTB-MBS MEF cells stably expressing RIDR construct to ascertain the identity of the observed RNA granules. *ACTB-MBS* FISH: magenta; SG marker G3BP1 IF: cyan; P-body marker DDX6 IF: green; DAPI: blue. **a**) After 30 minutes Rapa induction, there are ACTB-MBS mRNA granules, but no stress granule formation. **b**) The G3BP1 antibody label the stress granule after cells were treated with 1 mM Sodium Arsenite for 10 minutes, demonstrating the efficacy of G3BP1 antibody. **c)** After 30 minutes Rapa treatment in addition to 30-min treatment with 200µM Arsenite, the induced RNA granules did not colocalize with stress granules, but were seen near stress granules. **d)** After 30 minutes Rapa treatment, the induced RNA granules colocalize with P-body marker, DDX6. **e)** After 30 minutes Rapa treatment, the induced RNA granules colocalize with P-body marker, XRN1. White boxes were enlarged on the right for each. Scale bars: 5 µm for original images, 1 µm for zoomed images.

**Supplementary Figure 4**

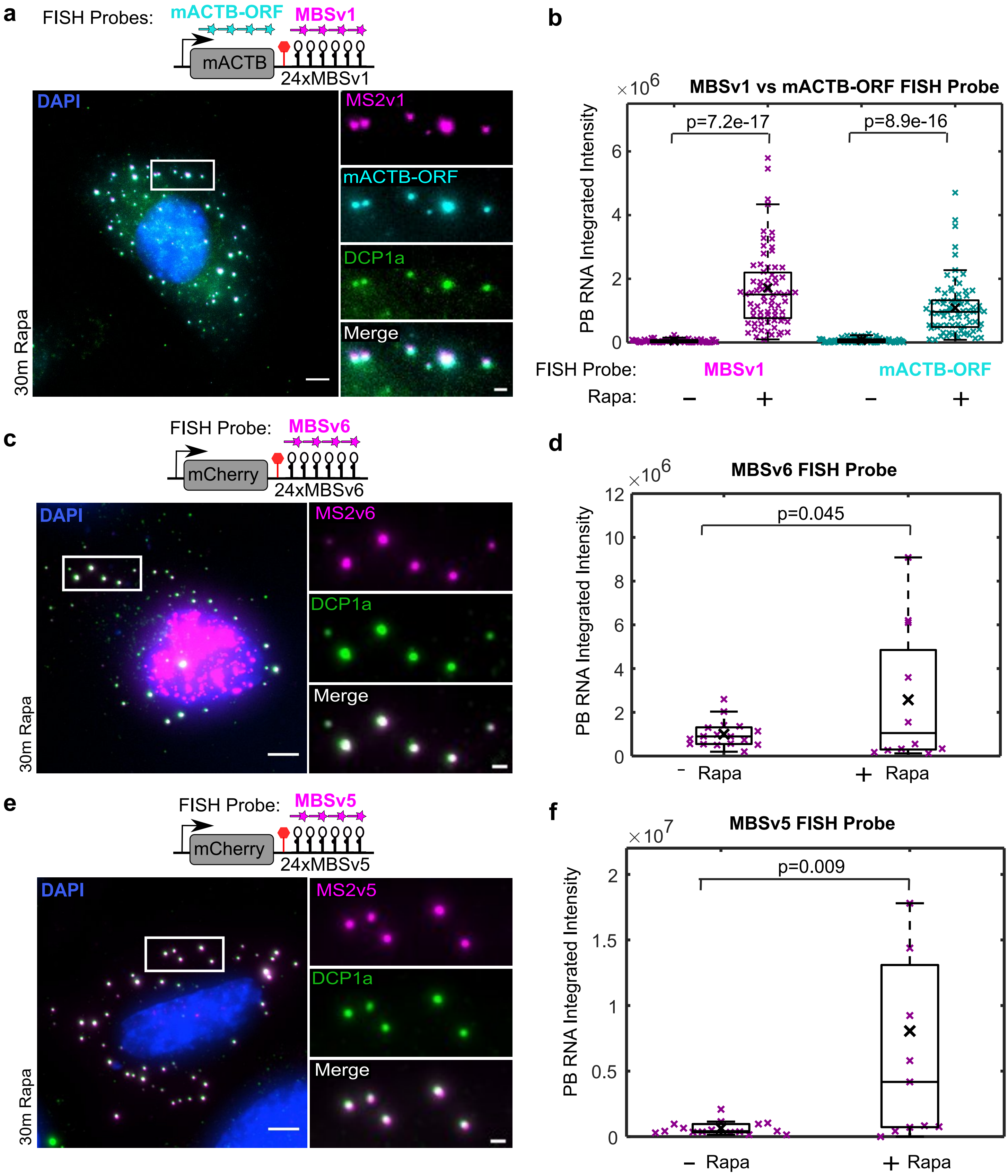

**Supplementary Figure 4:** ***ACTB-ORF and MBSv6 RNA are recruited to P-bodies.*** smFISH-IF experiments were conducted in ACTB-MBS MEF cells **a-b)** or U-2 OS cells transiently transfected with reporter mRNAs and RIDR construct (**c-f**). **a**) Representative ACTB-MBS MEF cell stably expressing RIDR construct. magenta: MBS FISH; cyan: ACTB-ORF FISH;green: P-body marker DCP1a IF; blue: DAPI. After 30 minutes Rapa induction, there were RNA granules containing both MBS and ACTB-ORF mRNAs that colocalized with P-bodies. **b**) The smFISH signal intensity in P-bodies was quantified and plotted for both sets of probes before and after treatment of 100nM Rapa for 30 minutes (63, 75 cells for -/+ Rapa, respectively, each from one independent experiment). **c,e)** U-2 OS cell transiently transfected with RIDR and reporter mCherry-24xMBSv6 (**c**) or mCherry-24xMBSv5 (**e**) RNA. Magenta: MBSv6 or MBSv5 FISH; green: P-body marker DCP1a IF; blue: DAPI. After 30 minutes Rapa induction, there were RNA granules formed in P-bodies by both mCherry-24xMBS reporters. **d,f)** The FISH signal intensities for MBSv6 (17, 12 cells for -/+ Rapa, respectively, each from one independent experiment) (**d**) and MBSv5 (16, 11 cells for -/+ Rapa, respectively, each from one independent experiment) (**e**) in P-bodies were quantified before and after treatment with 100nM Rapa for 30 minutes. The averages of each dataset is marked with a large black “X White boxes were enlarged on the right for each.Scale bars: 5 µm for original images, 1 µm for zoomed images. Each box plot chart displays the following information: the median as the center line, the lower and upper quartiles indicated by whiskers, and outliers indicated by individual data points outside the boxes. P-values from two-sample t-tests are above each comparison. Source data are provided as a Source Data file.

## Supplementary Figure 5

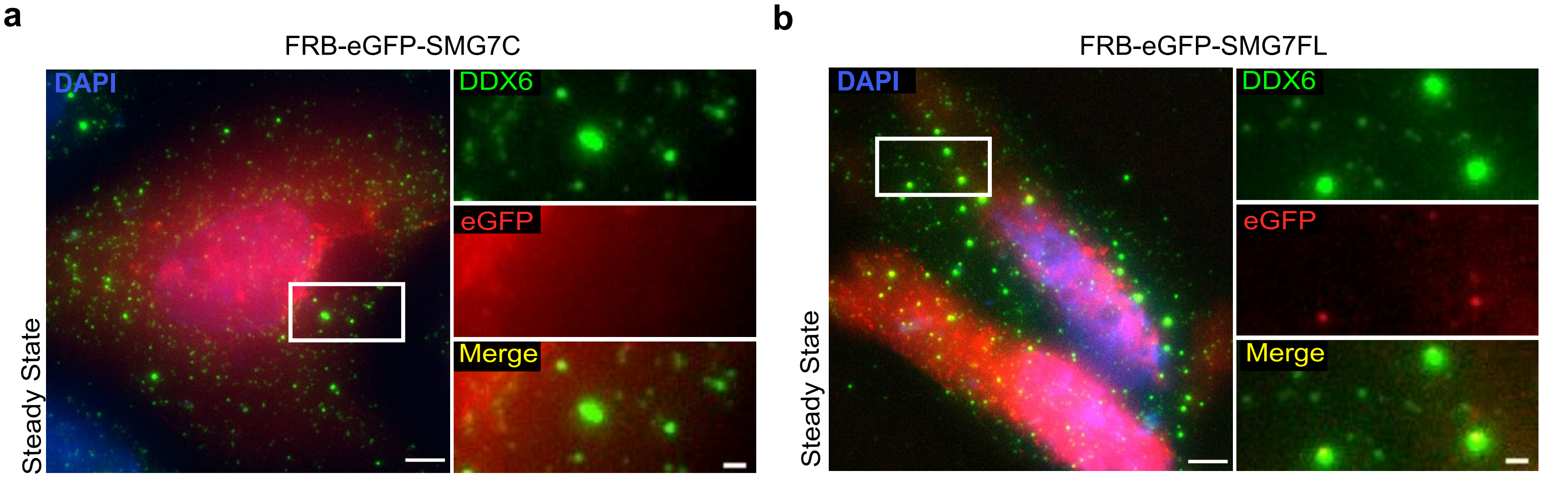

**Supplementary Figure 5:** ***SMG7C does not localize to P-bodies in steady state conditions.*** **a-b)** Representative IF images of U-2 OS cells transiently transfected with FRB-eGFP-SMG7C (**a**) or FRB-eGFP-SMG7FL (**b**) at steady state conditions. Number of cells inspected was 14 and 12, respectively, in one independent experiment. The white boxes were enlarged on the right. Green: DDX6 IF; red: eGFP; blue: DAPI. Scale bars: 5 µm for original and. 1 µm for zoomed images.

# Supplementary Figure 6

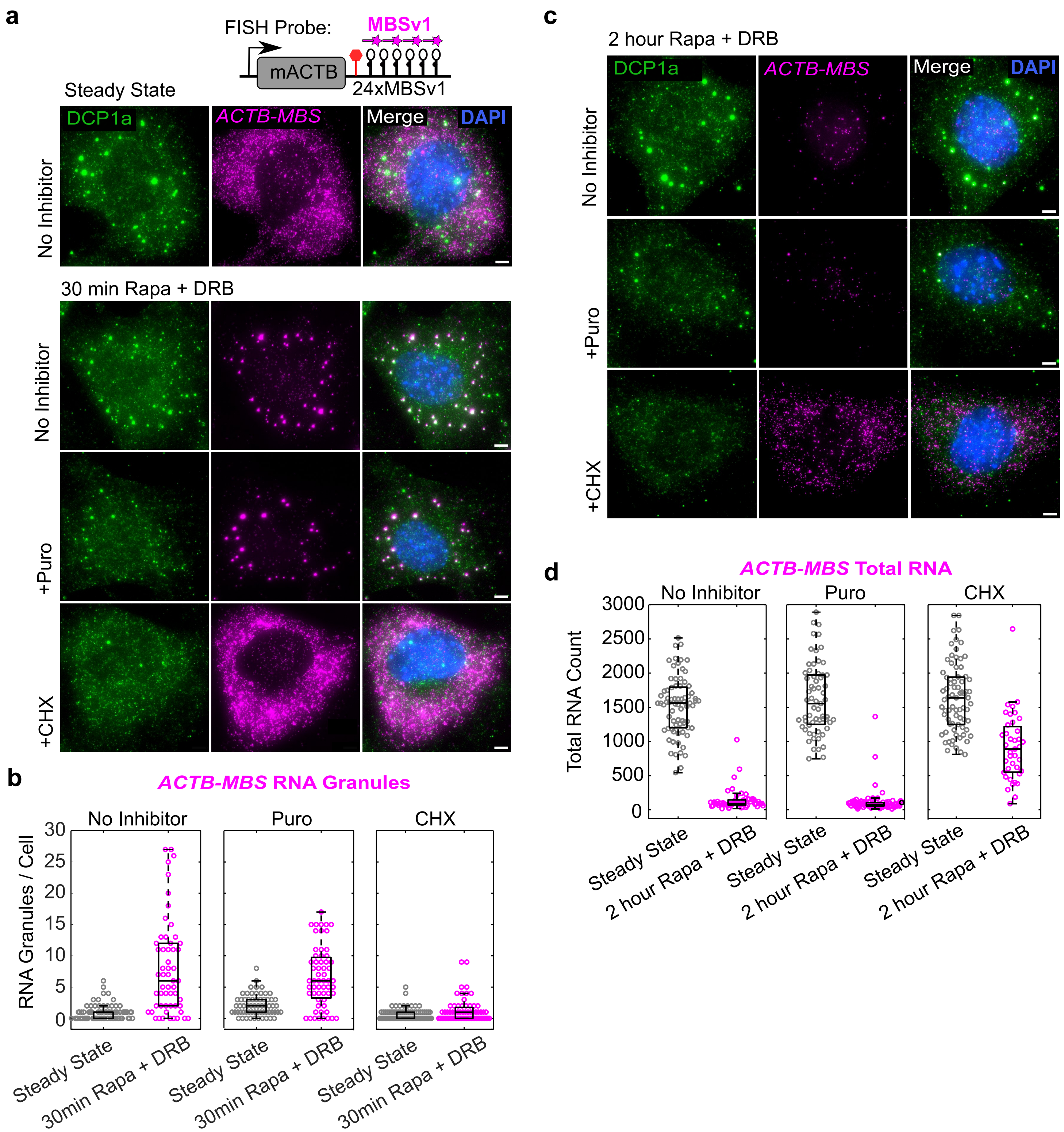

**Supplementary Figure 6: *Translation inhibitors have different effects on RNA decay dynamics.*** Different Translation inhibitors are combined with RIDR to study effect on RNA decay behavior **a)** Representative smFISH-IF images of ACTB-MBS MEF cells expressing RIDR construct then treated with no inhibitor, puromycin, or cycloheximide for 30 mins, followed by induction by rapamycin plus DRB for 30 min **b)** Quantification of *ACTB-MBS* RNA granule counts in Steady State vs 30 min Rapa + DRB after no translation inhibition, or treatment with puromycin or cycloheximide. **c)** Representative FISH-IF images of ACTB-MBS MEF cells expressing RIDR construct then treated with no inhibitor, puromycin, or cycloheximide for 30 mins, followed by induction by rapamycin plus DRB for 2 hours **d)** Quantification of (c) showing *ACTB-MBS* RNA counts in Steady State vs 2-hour Rapa + DRB after no translation inhibition, or treatment with puromycin or cycloheximide. 39-84 cells were quantified per condition in one independent experiment (the precise number of cells per condition are given in the source data). Scale bars: 5 µm. Each box plot chart displays the following information: the median as the center line, the lower and upper quartiles indicated by whiskers, and outliers indicated by individual data points outside the boxes. Source data are provided as a Source Data file.

## Supplementary Figure 7

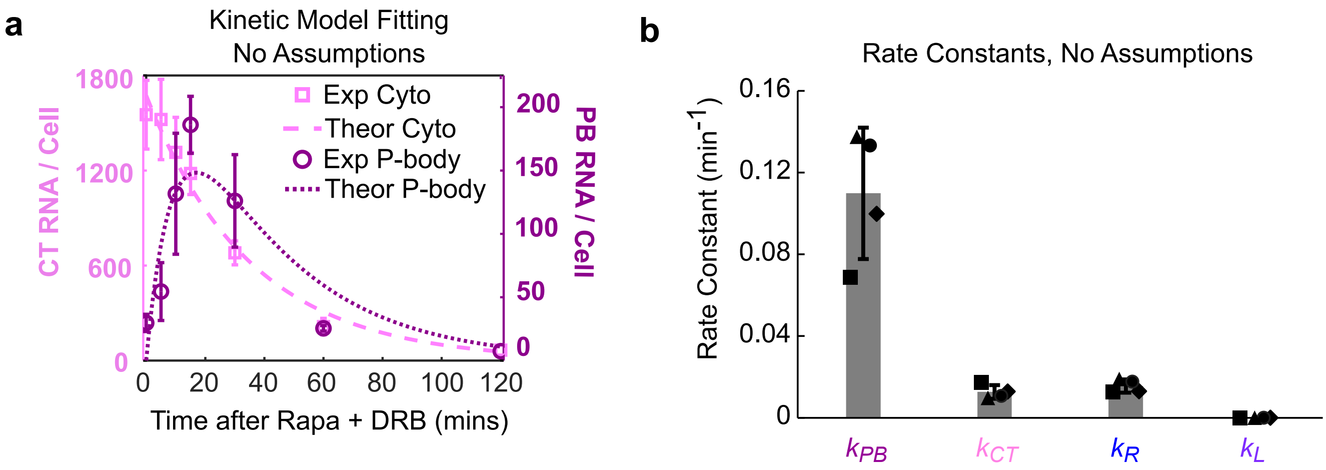

**Supplementary Figure 7:** ***Fitting to kinetic model with no assumptions shows similar results as Assumption III: $k_L$=0*** **a)** Fitting results with no assumptions. RNA counts in the P-body: dark magenta; RNA counts in the cytoplasm: light magenta; Experimental data: symbols; Theoretical fit: lines. Error bars represent standard deviation of the means of 4 replicates. **b)** Model parameters determined from fitting with no assumptions. Data are presented as mean values of the fitted parameters across the 3-4 biological replicates. Error bars represent the standard deviation. Source data are provided as a Source Data file.

# Supplementary Figure 8

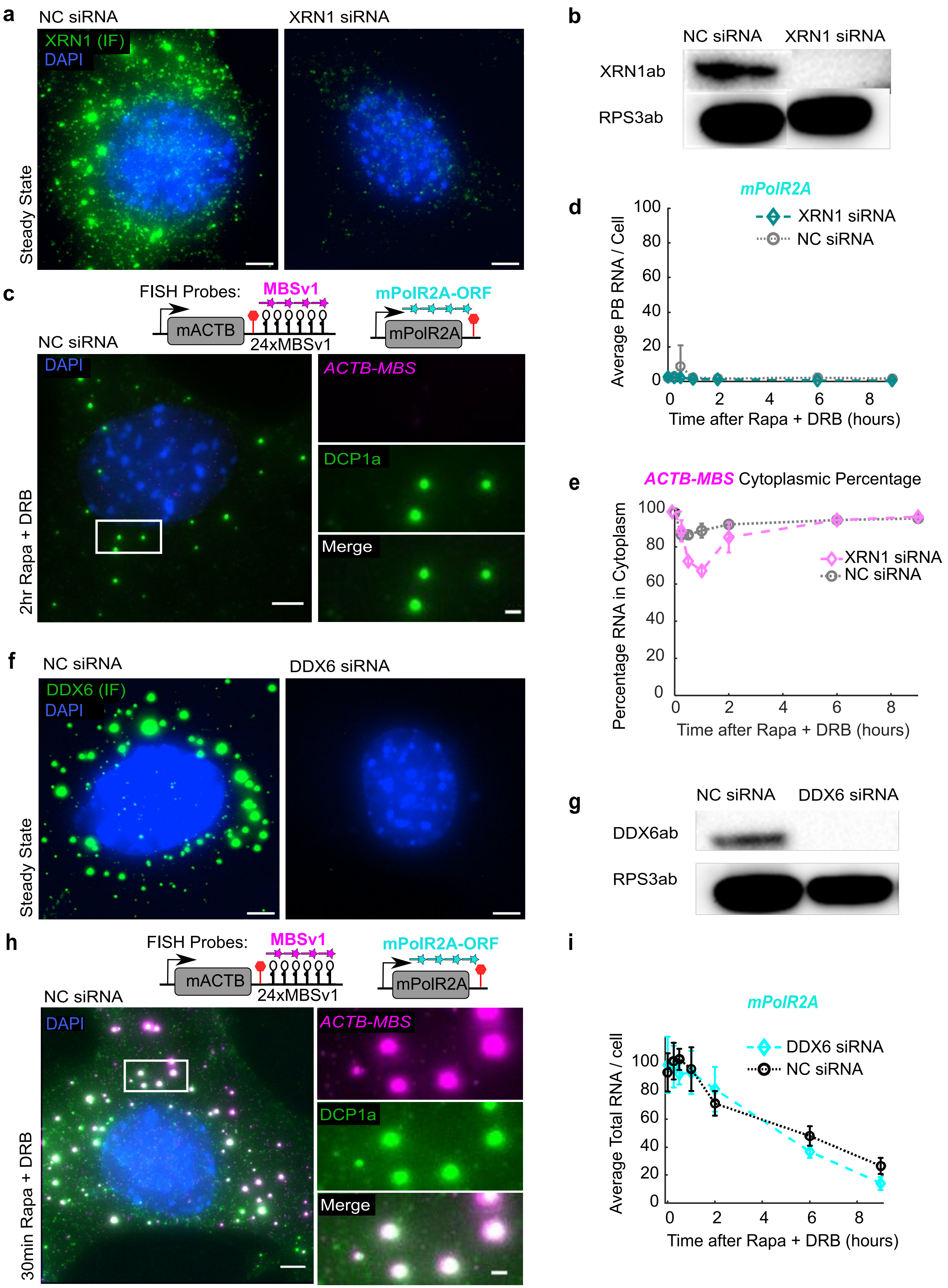

**Supplementary Figure 8.** ***Knockdown of DDX6 and XRN1 RNA is efficient****.* ACT-MBS MEF cells were treated with siRNA against XRN1 (**a-d),** DDX6 (**e-h**) or scrambled siRNA (NC) **(a-h**) for 72 hours. Cells were treated with Rapa and DRB, and fixed at different time points. **a)** Representative IF images for cells treated with NC (left) or XRN1 (right) siRNA. XRN1 IF: green; DAPI: blue. **b)** Western blots with XRN1 antibody on cells treated with NC (left column) or XRN1 (right column) siRNA with RPS3 antibody as an internal control. **c)** Representative smFISH-IF image for XRN1 siRNA treated cell at 2 hours post-induction. The white box was enlarged on the right. *ACTB-MBS* FISH: magenta; DCP1a IF: green; DAPI: blue. **d)** Quantification of mPolR2A mRNAs in P-bodies over 9-hour time course after induction. XRN1 siRNA: cyan; NC siRNA: gray. **e)** *ACTB-MBS* cytoplasmic mRNA as a percentage of total *ACTB-MBS* mRNA over 9-hour RIDR time course for cells treated with siRNAs of XRN1 (light pink) or NC (gray). **f)** Representative IF images for cells treated with NC (left) or DDX6 (right) siRNA. DDX6 IF: green; DAPI: blue. **g)** Western blots with DDX6 antibody on cells treated with NC (left column) or DDX6 (right column) siRNA with RPS3 antibody as an internal control. **h)** Representative smFISH-IF image for DDX6 siRNA treated cell at 30 minutes post-induction. The white box was enlarged on the right. *ACTB-MBS* FISH: magenta; DCP1a IF: green; DAPI: blue. **i)** Quantification of total *mPolR2A* mRNAs per cell over 9-hour time course after induction. DDX6 siRNA: cyan; NC siRNA: black. Error bars represent standard deviation of the means of 3-4 biological replicates. 193-515 cells were quantified per condition across replicates (the precise number of cells per condition per replicate are given in the source data). Scale bars: 5 µm for original images, 1 µm for zoomed images. Source data are provided as a Source Data file.

## Supplementary Figure 9

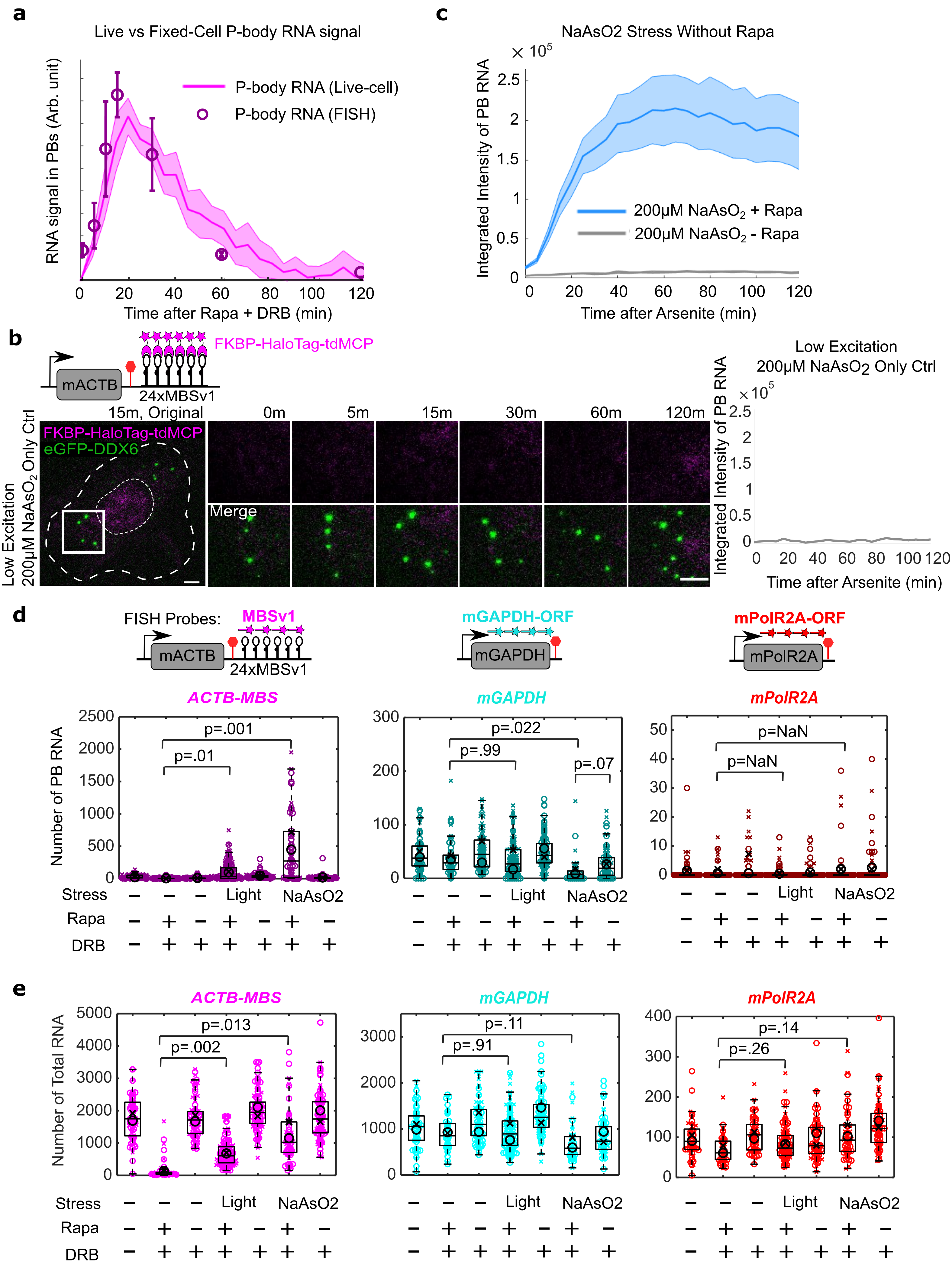

**Supplementary Figure 9.** ***Arsenite stress alone does not result in RNA accumulation in P-bodies.*** Live-cell imaging experiments were performed to track P-bodies (DDX6-eGFP, green) and *ACTB-MBS* (FKBP-HaloTag-tdMCP, magenta) after Rapa induction, with or without arsenite**.** **a)** Overlay of RNA signal in P-bodies over time after Rapa + DRB treatment determined from live-cell measurements under low laser power (light magenta with shaded error bars) versus fixed-cell smFISH-IF measurements (dark magenta). **b)** Representative movie montages under low excitation laser power and 200 µM (blue) NaAsO2 without Rapa **(Supplementary Movie 5);** The raw intensity trace of RNA intensity in P-bodies was shown on the right (gray line). Scale bars: 5 µm for both original and zoomed images. **c)** Raw RNA intensity in P-bodies after treatment with 200 µM NaAsO2 with (blue) and without (gray) Rapamycin (number of cells: 12, 13, respectively). Shaded error bars represent standard error. **(d-e)** ACTB-MBS MEF cells were treated with Rapa under different stress condition. The light stress was achieved using a LCI stimulator for 2 hours (Methods). The arsenite treatment was 200 µM $NaAsO_2$ for 2 hours. Multicolor smFISH-IF was performed to measure the ACTB-MBS and endogenous mRNA levels. RNAs in P-body (**d**) or total RNAs (**e**) for *ACTB-MBS* (magenta), or endogenous *mGAPDH* (cyan), *mPolR2A* (red) mRNAs under different stress conditions were quantified.56-134 cells were quantified per condition across 2 biological replicates(the precise number of cells per condition per replicate are given in the source data). Each box plot chart displays the following information: the median as the center line, the lower and upper quartiles indicated by whiskers, and outliers indicated by individual data points outside the boxes. The averages of each replicate are marked with a large "X" or "O". The medians of the 2 replicates were compared using a two-sample t-test and p-values are displayed above each comparison (p=NaN is the result of comparing zero values). Source data are provided as a Source Data file.
