# Supplementary material for: A rapid inducible RNA decay system reveals fast mRNA decay in P-bodies
Source: Nat Commun. 2024 Mar 28;15:2720. doi: 10.1038/s41467-024-46943-z (PMC10979015; doi:10.1038/s41467-024-46943-z)
Supplement: Supplementary file 2 — Reporting Summary [file 41467_2024_46943_MOESM2_ESM.pdf]

nature portfolio

Corresponding author(s): Bin Wu

Last updated by author(s): 2/12/2024

Reporting Summary

Nature Portfolio wishes to improve the reproducibility of the work that we publish. This form provides structure for consistency and transparency in reporting. For further information on Nature Portfolio policies, see our Editorial Policies and the Editorial Policy Checklist.

Statistics

For all statistical analyses, confirm that the following items are present in the figure legend, table legend, main text, or Methods section.

n/a	Confirmed	
☐	☒	The exact sample size (n) for each experimental group/condition, given as a discrete number and unit of measurement
☐	☒	A statement on whether measurements were taken from distinct samples or whether the same sample was measured repeatedly
☐	☒	The statistical test(s) used AND whether they are one- or two-sided<br>*Only common tests should be described solely by name; describe more complex techniques in the Methods section.*
☒	☐	A description of all covariates tested
☒	☐	A description of any assumptions or corrections, such as tests of normality and adjustment for multiple comparisons
☐	☒	A full description of the statistical parameters including central tendency (e.g. means) or other basic estimates (e.g. regression coefficient) AND variation (e.g. standard deviation) or associated estimates of uncertainty (e.g. confidence intervals)
☐	☒	For null hypothesis testing, the test statistic (e.g. F, t, r) with confidence intervals, effect sizes, degrees of freedom and P value noted<br>*Give P values as exact values whenever suitable.*
☒	☐	For Bayesian analysis, information on the choice of priors and Markov chain Monte Carlo settings
☒	☐	For hierarchical and complex designs, identification of the appropriate level for tests and full reporting of outcomes
☒	☐	Estimates of effect sizes (e.g. Cohen's d, Pearson's r), indicating how they were calculated

Our web collection on statistics for biologists contains articles on many of the points above.

Software and code

Policy information about availability of computer code

Data collection	Data imaging was performed using Nikon Elements v4.8. Flow Cytometry data was acquired using Thermo Attune NxT. Western blotting data was acquired by BIO-RAD ChemiDoc imaging system.
Data analysis	Single-molecule FISH data were analyzed using Matlab R2019b, FISH-Quanta v3, and uLocalize (https://doi.org/10.5281/zenodo.7922686). Analysis of live cell imaging was done using u-track v2, visualization and processing of the images was done using ImageJ v1.53t, MATLAB R2019b was used for plotting and data visualization, FlowJo_v10.6.1 was used to visualize and quantify flow cytometry data.

For manuscripts utilizing custom algorithms or software that are central to the research but not yet described in published literature, software must be made available to editors and reviewers. We strongly encourage code deposition in a community repository (e.g. GitHub). See the Nature Portfolio guidelines for submitting code & software for further information.

Data

Policy information about availability of data

All manuscripts must include a data availability statement. This statement should provide the following information, where applicable:

- Accession codes, unique identifiers, or web links for publicly available datasets
- A description of any restrictions on data availability
- For clinical datasets or third party data, please ensure that the statement adheres to our policy

Data Availability Statement: The data supporting the findings of this study are available from the corresponding authors upon reasonable request. Source data are provided with this paper.

Human research participants

Policy information about studies involving human research participants and Sex and Gender in Research.

Reporting on sex and gender	N/A
Population characteristics	N/A
Recruitment	N/A
Ethics oversight	N/A

Note that full information on the approval of the study protocol must also be provided in the manuscript.

Field-specific reporting

Please select the one below that is the best fit for your research. If you are not sure, read the appropriate sections before making your selection.

☒ Life sciences ☐ Behavioural & social sciences ☐ Ecological, evolutionary & environmental sciences

For a reference copy of the document with all sections, see nature.com/documents/nr-reporting-summary-flat.pdf

Life sciences study design

All studies must disclose on these points even when the disclosure is negative.

Sample size	No sample size calculation was performed for this work. For FISH/FISH-IF experiments (Figures 1-4) sample sizes were determined based on standard protocols in the field for these assays (Dave et al, Mol Cell 2023). For live-cell imaging, we completed 2-3 biological replicates per condition over a 2 hour time course. ~15-20 cells expressing both the RIDR and eGFP-DDX6 constructs were selected to be imaged over the 2 hour imaging per condition. At the end of the 2-hours, all cells that did not die, divide, or move out of the field of view were ultimately quantified.
Data exclusions	No data were excluded.
Replication	2-4 biological replicates were used, except for Supplemental Figures 4 - 6. The specific number of replicates and cells in each is listed in the figure legends.
Randomization	Cells were plated randomly to multi-well dishes, they were assigned randomly to different experimental group. The images were taken at random positions.
Blinding	The data collection was not performed blindly, as our image processing pipeline did not have subjective measurements. Selection of cells for imaging was done by choosing cells positive for FKBP-Halo-tdMCP, not by checking the RNA channels which were ultimately quantified.

Reporting for specific materials, systems and methods

We require information from authors about some types of materials, experimental systems and methods used in many studies. Here, indicate whether each material, system or method listed is relevant to your study. If you are not sure if a list item applies to your research, read the appropriate section before selecting a response.

Materials & experimental systems

n/a	Involved in the study	
☐	☒	Antibodies
☐	☒	Eukaryotic cell lines
☒	☐	Palaeontology and archaeology
☒	☐	Animals and other organisms
☒	☐	Clinical data
☒	☐	Dual use research of concern

Methods

n/a	Involved in the study	
☒	☐	ChIP-seq
☐	☒	Flow cytometry
☒	☐	MRI-based neuroimaging

Antibodies

Antibodies used	Primary antibodies: DCP1a (1:1000 for IF, Abcam ab183709), XRN1 (1:100 for IF, 1:1000 for WB, Bethyl Laboratories A300-443A-M), DDX6 (1:1000 for IF, 1;10000 for WB, Bethyl Laboratories A300-461A), G3BP (1:100 for IF, Aviva Systems Biology ARP37713_T100), RPS3 (1:250 for WB, Santa Cruz sc-376008). Secondary Antibodies: Goat anti-rabbit (1:5000 for IF goat-anti-rabbit IgG (H+L) Alexa Fluor 750, Invitrogen A-21039), Mouse anti-rabbit IgG HRP (1:5000 for WB Santa Cruz sc-2357)
Validation	All antibodies used in this work are commercially available and the validation can be accessed at manufactures website. We validated these antibodies in our own hands using immunofluorescence and western blotting. We validated DDX6 and XRN1 using siRNA against these genes, then used immunofluorescence and western blot assays to confirm knockdown (Supplemental Figure 8). DCP1a antibody was validated by immunofluorescence which showed characteristic targeting of P-body structures (Figure 2) and according to the manufacturer's website it was also knockout validated. G3BP was validated in our own hands by comparing the labeling of stress granules with and without sodium arsenite stress (Supplementary Figure 3), and according to the manufacturer's website, all Aviva antibodies have been rigorously validated, though specific methods were not listed for this particular antibody. The RPS3 primary antibody used as a loading control for the western blot experiments was validated via knock down in Hegde V, et al. DNA Repair (2007) PMID: 17049931.

Eukaryotic cell lines

Policy information about cell lines and Sex and Gender in Research

Cell line source(s)	U-2 OS (American Type Culture Collection HTB-96); HEK293T (American Type Culture Collection CRL-1573); ACTB-MBS MEF (a gift from Dr. Robert Singer's lab, published in Lionett et al. Nature Methods, 2011. PMID: 21240280)
Authentication	U-2 OS and HEK293T cells were from commercial resources, which were validated with karyotyping by members of our lab. ACTB-MBS MEF cells were from a mouse embryo primary cell line and were previously published in Lionett et al. Nature Methods, 2011. PMID: 21240280. Within this publication, PCR and RNA FISH were used to verify that the MS2 stem loops were added to each ACTB locus.
Mycoplasma contamination	Cell lines were tested monthly for mycoplasma and were consistently negative.
Commonly misidentified lines (See ICLAC register)	No commonly misidentified cell lines were used in the study

Flow Cytometry

Plots

Confirm that:

☒ The axis labels state the marker and fluorochrome used (e.g. CD4-FITC).

☒ The axis scales are clearly visible. Include numbers along axes only for bottom left plot of group (a 'group' is an analysis of identical markers).

☐ All plots are contour plots with outliers or pseudocolor plots.

☒ A numerical value for number of cells or percentage (with statistics) is provided.

Methodology

Sample preparation	Cells were dissociated and resuspended in DMEM and analyzed by FACS.
Instrument	Attune NxT (Thermo)
Software	FlowJo (version 10.1.6, FlowJo LLC)
Cell population abundance	Between 1000-3000 Halo-Tag positive cells were analyzed by FACS per condition and mCherry reporter expression was determined by YL2-H signals.
Gating strategy	The live, single cells were first gated using FSC-A/SSC-A. mCherry-MBS expressing cells were gated using YL2-H. JF503 (Halo)

ligand was gated using BL1-H. Cells sufficiently above blank control in BL1-H were measured for geometric mean The cells without rapamycin were used as negative control for the inducible tethering experiments. In the case of direct tethering, the cells without a decay factor were used as a negative control.

☒ Tick this box to confirm that a figure exemplifying the gating strategy is provided in the Supplementary Information.
