# Supplementary material for: A rapid inducible RNA decay system reveals fast mRNA decay in P-bodies
Source: Nat Commun. 2024 Mar 28;15:2720. doi: 10.1038/s41467-024-46943-z (PMC10979015; doi:10.1038/s41467-024-46943-z)
Supplement: Supplementary file 4 — Description of Additional Supplementary Files [file 41467_2024_46943_MOESM4_ESM.pdf]

Supplementary Movie 1. *Live-cell imaging of ACTB-MBS MEF cells showing RNA granule formation.* Live-cell imaging was done over 2 hours to visualize *ACTB-MBS* (FKBP-HaloTag-tdMCP, magenta). This cell was imaged at 5-minute time intervals using low excitation laser power. *ACTB-MBS* RNA granules can be observed within 10 minutes and disappear within 1 hour. Scale bar: 5 µm.

Supplementary Movie 2. *Live-cell imaging of ACTB-MBS MEF cells with high excitation conditions.* Live-cell imaging was performed to track P-bodies (DDX6-eGFP, green, left) and *ACTB-MBS* (FKBP-HaloTag-tdMCP, magenta, middle) to assess their colocalization (merged image, right) after induction. This is the full movie of the montages displayed in **Fig. 5a** showing a representative ACTB-MBS MEF cells treated with Rapa then imaged at 5-minute time intervals with high excitation laser power. Scale bar: 5 µm.

Supplementary Movie 3. *Live-cell imaging of ACTB-MBS MEF cells with low excitation conditions.* Live-cell imaging was performed to track P-bodies (DDX6-eGFP, green, left) and *ACTB-MBS* (FKBP-HaloTag-tdMCP, magenta, middle) to assess their colocalization (merged image, right) after induction. This is the full movie of the montages displayed in **Fig. 5b** showing a representative ACTB-MBS MEF cells treated with Rapa then imaged at 5-minute time intervals with the minimal laser power sufficient to observe RNA granules colocalized with P-bodies. Scale bar: 5 µm.

Supplementary Movie 4. *Live-cell imaging of ACTB-MBS MEF cells with low excitation conditions after 30-minute pre-treatment with 200µM Sodium Arsenite.* Live-cell imaging was performed to track P-bodies (DDX6-eGFP, green, left) and *ACTB-MBS* (FKBP-HaloTag-tdMCP, magenta, middle) to assess their colocalization (merged image, right) after induction. This is the full movie of the montages displayed in **Fig. 5c** showing a representative ACTB-MBS MEF cells treated with Rapa then imaged at 5-min time intervals with minimal laser power after cells were pretreated with 200 µM Sodium Arsenite for 30 minutes. Scale bar: 5 µm.

Supplementary Movie 5. *Live-cell imaging of ACTB-MBS MEF cells treated with 200µM Sodium Arsenite, but without adding Rapamycin.* Live-cell imaging was performed to track P-bodies (DDX6-eGFP, green, left) and *ACTB-MBS* (FKBP-HaloTag-tdMCP, magenta, middle) to assess their colocalization (merged image, right) after induction. This is the full movie of the montages displayed in **Fig. S9b** showing a representative ACTB-MBS MEF cells treated with 200 µM Sodium Arsenite then imaged with minimal laser power at 5-min time intervals. Scale bar: 5 µm.

Supplementary Data 1, siRNA sequences

siRNA sequences ordered from IDT

Supplementary Data 2, FISH Probe sequences

MBSv5 smFISH probes

hPolR2A smFISH probes

MBSv1 smFISH probes

mPolR2A smFISH probes

mACTB-ORF smFISH probes

MBSV6 smFISH probes

mGAPDH smFISH probes
